# Injection versus Decompression for Carpal Tunnel Syndrome-Pilot trial (INDICATE-P)—protocol for a randomised feasibility study

**DOI:** 10.1186/s40814-017-0134-y

**Published:** 2017-04-24

**Authors:** Will Mason, Daniel Ryan, Asif Khan, Hui-Ling Kerr, David Beard, Jonathan Cook, Ines Rombach, Cushla Cooper

**Affiliations:** 1 Gloucestershire Royal Hospital, Gloucestershire Hospitals NHS Foundation Trust, Gloucester, England; 2 Bristol Royal Infirmary, University Hospitals Bristol NHS Foundation Trust, Bristol, England; 3 Royal College of Surgeons Surgical Intervention Trials Unit, NDORMS, University of Oxford, Oxford, UK; 4 Centre for Statistics in Medicine, Royal College of Surgeons Surgical Intervention Trials Unit, NDORMS, Oxford, UK

**Keywords:** Carpal tunnel syndrome, Steroid injection, Carpal tunnel decompression, Randomised controlled trial

## Abstract

**Background:**

Carpal tunnel syndrome (CTS) is the commonest peripheral nerve disorder in the UK, with over 52,996 carpal tunnel decompressions performed in 2011. By 2030, this figure is estimated to double. Whilst evidence supports conservative measures for mild symptoms, and early surgery for severe symptoms, controversy remains over the most appropriate management for patients that present with moderate disease, with regard to early surgery or late surgery following steroid injection. Injection versus Decompression for Carpal Tunnel Syndrome-Pilot trial (INDICATE-P) is a feasibility study for a multicentre, randomised controlled trial (INDICATE) to determine whether patients over the age of 18 with moderate CTS should undergo early surgical decompression of the median nerve or a single steroid injection (followed by later surgery if required).

**Methods/design:**

INDICATE-P is a feasibility study for an open (non-blinded) randomised controlled pilot trial. Eligible participants will be adults with a clinical diagnosis of moderate CTS. This is defined as symptoms disturbing sleep or restricting activities of daily living or work, despite a 2-week trial of night splints. Participants will be randomised to one of two possible interventions: surgical decompression or a single steroid injection (followed by surgery later if required). Clinical outcome measures will be captured by postal questionnaire at 1, 3, 6 and 12 months post-randomisation. In order to improve the study design for the main INDICATE trial, feasibility data will also be collected to identify difficulties in recruitment and retention, to gain patient feedback on questionnaires and to confirm the suitability of the proposed outcome measures.

**Discussion:**

The INDICATE-P feasibility study will contribute to the design and execution of the INDICATE trial, which will seek to assess the safety and effectiveness of two approaches to treatment for patients over 18 years of age with moderate CTS: early carpal tunnel decompression or a single steroid injection (followed by later surgery).

## Background

Carpal tunnel syndrome (CTS) is the most common chronic hand condition referred for surgery. Incidence of surgery for CTS is increasing worldwide [1–4]. The UK has seen a 34% increase in the number of carpal tunnel decompression surgeries between 1998 and 2015, with the number of operations in 2030 estimated to be 105,000 per year: double the number in 2011 [4].

Whilst patients with mild CTS can improve both clinically and neurophysiologically [5–7], the majority of cases eventually increase in severity over time [8].

The treatment of CTS depends on severity of symptoms. Severe CTS is defined as presence of thenar muscle wasting and constant numbness. Mild CTS should be treated non-operatively, whilst moderate CTS can be treated either non-operatively (wrist splinting or steroid injecting [9–12]) or with surgery [13]. There is no consensus as to the clinical distinction between mild and moderate CTS or how the latter should be treated [14].

### Treatment

Steroid injections provide a good initial response in around 70–90% of patients with mild and moderate CTS, but relapse is common. The duration of effect reported in the literature varies, but one study showed that 63% of patients had a sustained effect after 6 months, but only 34% after 18 months [15]. The risks of injection are small, with the incidence of median nerve injury from intra-neural injection estimated to be <0.1% in competent hands [16]. There is no evidence to guide treatment following relapse after steroid injection, though if the duration of effect was reasonable, some patients prefer to have further injections and avoid surgery [12].

Carpal tunnel decompression (CTD) is 90–95% effective in permanently relieving pain, paraesthesia and intermittent numbness in CTS [17, 18]. However, it involves higher risk and longer recovery time for the patient than steroid injection. A survey of 4000 patients who received CTD found that 2 years after surgery, only 75% considered the operation a success and 8% were worse off [19]. This study shows a clear trend to improved results in patients with moderate CTS compared to mild and severe CTS.

There is variability in Clinical Commissioning Group (CCG) policies and clinician approaches: this stems from the paucity of good evidence. Only two randomised controlled trials have compared steroid injections to surgery. Ly-Pen et al. [18], across 163 wrists, found no significant difference 2 years, but no validated symptom score was used, and they found high cross-over between treatment groups. Hui conducted a trial of 50 patients, finding that surgery provided greater relief of symptoms at 20 weeks [20]. A 2008 Cochrane review [21] suggests that it is unclear whether surgery is superior to steroid injection.

For patients with moderate CTS, the dilemma of whether to opt for surgery or steroid injection remains. The INDICATE study will seek to assess the clinical and cost effectiveness of the two approaches to treatment; early surgery or a single steroid injection (with later surgery if required).

## Methods/design

Injection versus Decompression for Carpal Tunnel Syndrome-Pilot trial (INDICATE-P) is a pilot study to inform the design of the multicentre, randomised controlled INDICATE study, specifically in terms of feasibility of recruitment and data collection. The aim of the main trial is to assess whether early surgery or a single steroid injection (with later surgery if required) is the better treatment approach for patients with moderate CTS. The objectives of this feasibility study are as follows:

### Primary objective

To assess the feasibility of conducting the INDICATE trial with regards to recruitment, data collection and outcome measurement. In order to achieve this, a range of study process measures and clinical and patient-reported outcomes will be collected as listed below.

### Secondary objectives

1. Identify challenges and differences associated with recruiting from the primary and secondary care
2. Assess patient views on the follow-up method and data collection
3. Confirm suitability of outcome set for INDICATE trial

### Outcomes

Study process measures:

1. Number of potentially eligible patients identified in secondary and primary care units
2. Number of patients approached to take part in the study
3. Proportion of patients who consented to take part in the study (out of those approached)
4. Proportion of patients who received the allocated treatment and reasons for any non-compliance (out of those randomised)
5. Proportion of patients with a valid response at each follow-up time point (out of those randomised)
6. Assessing the process of establishing primary care recruitment portals, and the proportion of patients recruited via this process.

Baseline and follow-up questionnaires will contain a variety of patient-reported clinical outcomes that are candidates for use in the main INDICATE trial. The following will be collected from participants in both trial arms for comparison:

- Symptoms and function will be measured using Boston Carpal Tunnel Assessment Questionnaire [22]; this is a validated patient-orientated scale specific for CTS. It has two parts: a symptom severity scale (SSS) consisting of 11 questions and a function status scale (FSS) with 8 questions. Each question is scored from 1 to 5, and the mean is calculated for each subscale. The change in SSS score will constitute the primary outcome for the main trial (baseline 1, 3, 6 and 12 months).
- Pain will be measured using the palmar pain scale [23]; this is a validated scale for pain-related activity limitation (baseline 1, 3, 6 and 12 months).
- Patient satisfaction [24] will be assessed using a variation of the Oxford Satisfaction Index. This involves transition questions developed and utilised recently by members of the study team to measure patients’ satisfaction post treatment. (12 months)
- Information on time off work/activities (1, 3, 6 and 12 months)
- Standardised health outcomes will be measured using EuroQol-5D-3L (baseline 1, 3, 6 and 12 months)

Additionally we will also collect:

- details of other healthcare received related to the patient’s CTS
- complication data
- patient preference regarding postal or online questionnaires as part of study follow-up
- patient views on the study questionnaires and trial participations

Clinical outcomes:

- These are collected via the patient-reported questionnaires and the case report forms completed for the study interventions. Information collected includes:
  - Details on any complications (whether surgical or not)
  - Details on the procedure time, local anaesthetic used, antibiotics prescribed, operative findings, staff involved in the procedures and planned follow-up regimen are collected on the injection and surgery case report form (CRF) at the time of surgery.

### Adverse events

Adverse events (AE) will be captured primarily through patient-reported forms, but also by clinicians (either at participating centres or in primary care). AE’s will be reported by participating centres.

### Expected adverse events

The following is a list of events that are deemed “expected” in relation to these treatment approaches for moderate carpal tunnel syndrome:

- Surgical site infection—as defined using the Centre of Disease Control and Prevention Criteria
- Nerve injury—for either intervention, defined as permanently reduced median nerve sensibility or motor power
- Vessel injury—defined as a superficial palmar arch injury seen intra-operatively or a haematoma requiring drainage
- Complex regional pain syndrome (CRPS)—defined using the Budapest Criteria

### Recruitment

**Fig. 1:**
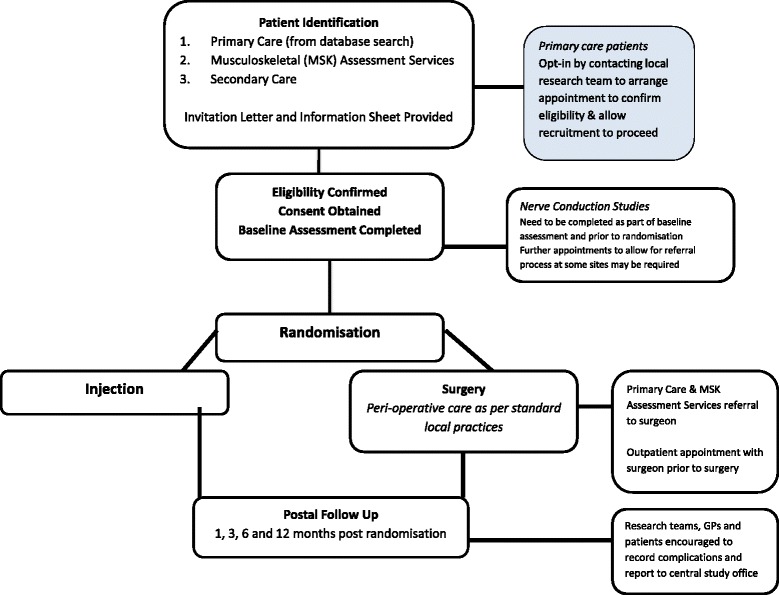
Patient recruitment flowchart

Participants will be recruited prospectively at the participating sites. The process of patient identification and recruitment will depend on the local treatment pathways at each participating site: from secondary care (Gloucester, Canterbury and Plymouth, UK) or from primary care using Patient Identification Centres at local General Practices (Gloucestershire, UK). Practices will identify patients by searching their databases on a monthly basis for patients with a new diagnosis of CTS. These patients will be sent a patient information sheet and a letter inviting them to contact the local research team in secondary care if they are interested in participating in the trial. The flowchart in Fig. 1 details the recruitment process.

### Informed consent

The patient must personally sign and date the latest approved version of the informed consent form before any study-specific procedures are performed.

Consent will be obtained before any study procedures are performed, including any required nerve conduction studies.

If an eligible patient does not wish to participate, patients will be asked if they would be willing to provide a reason for this. Patients are under no obligation to do so, but information like this will be utilised to inform the definitive trial.

With patient consent, GPs will be notified of their patient’s participation in the trial.

### Number of participants

A sample size of 40 patients will be used for this feasibility study. As this is a feasibility study, the study is not powered for pre-defined statistical tests. Instead, 40 participants are anticipated to be sufficient to enable a robust assessment of the objectives, i.e. the feasibility of a larger definitive trial, and also to provide a sufficiently robust estimate to inform a sample size calculation for the definitive trial.

### Study population

Patients over 18 years of age, who have moderate carpal tunnel syndrome, will be considered eligible for inclusion in this study. This includes patients who have tried night splints for at least 2 weeks, have disturbed sleep or have limited ability to perform activities of daily living (including work-related tasks). Nerve conduction studies will be conducted to provide an objective measure of electrophysiological severity and to allow analysis of their effectiveness as a prognostic indicator. Patients must be able to consent for themselves, have an understanding of the nature of the study and be willing to complete the follow-up requirements.

### Inclusion criteria

- All three of the following must be present:
  - O Intermittent paraesthesia predominantly within, but not exclusive to, the median nerve distribution
  - O Nocturnal hypoaesthesia, dysaesthesia or paraesthesia (including on waking)
  - O A positive provocation test (e.g. Tinel’s, Phalen’s, Durkan’s pressure or hand elevation test)
- Symptoms present for at least 3 months
- Patients’ symptoms must either:
  - O disturb their sleep or
  - O limit their ability to perform work or activities of daily living
- Patients must have failed a trial of night splints for at least 2 weeks
- Age >18 years

### Exclusion criteria

The participant may not enter the study if ANY of the following apply:

- Severe CTS
  - ○ thenar muscle wasting or
  - ○ continuously reduced light touch sensation in median nerve distribution (compared to opposite unaffected side or unaffected finger)
- Previous carpal tunnel surgery or steroid injection (either side)
- CTS secondary to:
  - ○ wrist deformity, trauma or mass
  - ○ pregnancy
  - ○ hypothyroidism
  - ○ inflammatory arthropathy
- Clinical or neurophysiological evidence of generalised or other peripheral neuropathy (e.g. ulnar nerve) or cervical radiculopathy (not based on NCS only performed for the purposes of the trial).
- Other symptomatic disorder in the affected hand diagnosed in the last 6 months or requiring treatment
- Patients in whom the baseline questionnaire cannot be completed due to cognitive difficulties

Patients with bilateral symptoms will have only one hand involved in the trial. This will be the hand with the more severe symptoms. The lesser-affected hand can be treated either by surgery or injection as the patient chooses, but treatment must take place *after the treatment* for the more severely affected hand.

### Interventions

Eligible and consenting patients will be randomised to receive one of the two possible treatment approaches:

#### Surgery

Surgeons treating INDICATE-P patients will regularly perform carpal tunnel decompression and will use the technique with which they are most familiar, whether it is open or endoscopic surgery, to avoid any learning curve effect.

Peri-operative management including anaesthesia, analgesia and dressings should follow local protocols. Post-operative management including dressing changes, advice, exercises, scar management and follow-up appointments should follow local protocols or the surgeon’s preference. Specifically, it will not be necessary to have a follow-up appointment in secondary care, if that is not usual local practice.

The designated investigator at the centre providing the surgery should ensure that the operative data is recorded on the appropriate CRF. The purpose of this form is to collect data on the type of surgery performed and key costs.

#### Steroid injection

All participants allocated to receive a steroid injection will receive the standard injection offered at their site. The technique of the injection will not be standardised, but the practitioner should regularly give injections for CTS and should use the technique with which they are most familiar. Details of this, along with the steroid and its dosage, will be recorded on the injection form.

Further injections are not advisable whilst the patient is in the study, however, if more than one injection is given, this needs to be recorded as further treatment on the complication form.

If symptoms recur following either the injection or surgery, participants should contact the local research team by email or by phone. Patients will then be referred to the centre of their choice to discuss further treatment options. This may include surgery as described above. Data about any surgery following injection will also be recorded on the appropriate CRF.

### Randomisation

A web-based randomisation system will be provided by the Oxford Clinical Trials Research Unit (OCTRU). The trial statistician will generate the random sequence, which will be simple block (with varying block size) randomisation, stratified by centre to ensure a similar number of patients are allocated to each treatment arm at each site. The research nurse will randomise participants once they have given informed consent and attended their baseline assessment. The randomisation system will automatically forward to the central study office in Oxford, and this will be sent to the nominated people at each site. The randomised treatment will be recorded on the site’s randomisation log. This log will be maintained by the local study team at each site.

Once a patient has been randomised, a copy of the patient’s consent form and the patient details form will be forwarded to the central study office in Oxford to facilitate the postal follow-up.

### Data collection

Patient demographics will be recorded on the patient details form. Data recorded will include date of birth, gender, hospital number, and the hand involved in the study.

Baseline data collected will include information about the patient’s pain and function, questions about their general health, duration of symptoms and employment status.

Neurophysiological testing (nerve conduction studies) are required as a baseline assessment. Results from these will be assessed by local staff using a standardised severity scale, anonymised and sent to Dr Jeremy Bland (co-investigator and neurophysiologist) at East Kent University Hospitals NHS Foundation Trust. He will assess the NCS reports and apply a severity grade to them according to the Bland Criteria [25].

If the research clinic is held in a hospital with a neurophysiology department, then ideally the nerve conduction studies will be scheduled at the same time as the baseline appointment to minimise inconvenience to the patient. If the research clinic is held in a hospital that does not have a neurophysiology department then the nerve conduction studies will need to be performed before the patient can be randomised. Specific consent for the studies will be given before they are performed. The patient may not need to return for another appointment, as the research team could proceed with randomisation and liaise with the patient via telephone about their randomised treatment and any further appointments that may be required. Extra appointments will be avoided where possible. A member of the research team will telephone the patient just prior to randomising to re-confirm that the patient still consents to take part.

### Follow-up assessments

Clinical outcome measure time points will be at baseline and 1, 3, 6, and 12 months post-randomisation.

Follow-up for study purposes will be completed via post. Clinical follow-up will occur as per routine practice at each participating centre.

### Blinding

INDICATE-P will be an open study where those delivering the care will not be blinded to the intervention nor will the patient who has received it or the outcome assessors.

### Analysis

A single analysis of data will take place once the study has ceased recruiting, and the last patient has reached their final assessment. No interim analysis is planned. Given this is a feasibility study, no formal statistical analysis of the data between groups is planned, i.e. no statistical tests for statistically significant differences in outcomes between trial arms will be performed. Descriptive analyses of outcome data will be carried out using appropriate summary measures (e.g. number of events and percentage for binary measures). Measures will be quantified, and where appropriate an associated 95% confidence interval calculated (e.g. using the Wilson score interval method (or an equivalent one) for binary measures). No imputation of missing data will be carried out.

### Patient recruitment data

The number of patients recruited per month will be presented by centre and overall. Reasons for ineligibility and non-participation where eligible will be summarised. The different centres participating in this pilot study represent different referral pathways that will aid in assessing the feasibility of each recruitment approach.

### Analysis of compliance

The compliance with the randomised intervention will be summarised as a proportion both overall and by treatment arm, together with reasons for non-compliance and withdrawals, where available. The time between randomisation and the trial intervention will also be summarised.

Completeness of data returns will be summarised by follow-up time point.

### Analysis of clinical and patient-reported outcomes

Outcome data will be summarised overall and according to the allocated intervention irrespective of the actual treatment received at baseline and during the follow-up time period. Results of nerve conduction studies will also be collected and categorised according to the Bland criteria [25].

Complications reported during the trial follow-up will also be summarised by allocated intervention.

## Discussion

CTS is the commonest peripheral nerve disorder in the UK and is increasing in incidence in the population, representing a significant disease burden. A lack of strong evidence regarding treatment has resulted in significant variability between both regions and individual clinicians, across a range of specialties.

INDICATE-P will inform design and methodology of the main INDICATE trial. The aim of the pilot study is to assess feasibility of recruitment and data collection, in particular, the number of eligible patients that present over the time period, the number who consent to participate, the number who receive the allocated treatment and complete follow-up, and also reasons for non-compliance. This information will aid in the practical development and application of INDICATE; a large, multicentre RCT that aims to determine whether steroid injection (with late surgery) or early surgery provides the best outcome for patients with moderate CTS.

### Trial status

The trial is currently recruiting.

## Abbreviations

CCG: Clinical Commissioning Group
CRF: Case report form
CTD: Carpal tunnel decompression
CTRG: Clinical Trials and Research Governance, University of Oxford
CTS: Carpal tunnel syndrome
GP: General practitioner
NHS: National Health Service
RCT: Randomised controlled trial
REC: Research Ethics Committee
